# Case Report: Primary low-grade dedifferentiated liposarcoma of the urinary bladder with molecular confirmation

**DOI:** 10.3389/fonc.2023.1221027

**Published:** 2023-10-10

**Authors:** Jian Cui, Ran Peng, Yahan Zhang, Yang Lu, Xin He, Min Chen, Hongying Zhang

**Affiliations:** Department of Pathology, West China Hospital, Sichuan University, Chengdu, China

**Keywords:** atypical lipomatous tumor/well-differentiated liposarcoma, dedifferentiated liposarcoma, urinary bladder, 12q13-15 amplification, MDM2 amplification, JUN amplification, whole exome sequencing

## Abstract

Liposarcomas originating in the urinary bladder are extremely rare. Only six cases of bladder liposarcoma have been reported, and all have been described as myxoid liposarcomas. Notably, none of the patients underwent molecular testing. Here, we report a dedifferentiated liposarcoma (DDL) that occurred in the urinary bladder, primarily in a 69-year-old Chinese woman, with infrequent low-grade dedifferentiation. Computed tomography (CT) revealed an ill-defined solid mass in the anterior bladder wall. The patient underwent a partial bladder resection. Histologically, the tumor cells with mild-to-moderate nuclear atypia were arranged in fascicular and storiform patterns, mimicking a low-grade fibroblastic tumor. In addition, scattered small foci of typical lipoma-like well-differentiated components were identified. Immunohistochemically, the tumor tested positivity for MDM2, CDK4, and p16. Fluorescence *in situ* hybridization revealed *MDM2* gene amplification in the neoplastic cells. Whole-exome sequencing showed that this tumor also harbored *CDK4*, *TSPAN31*, and *JUN* amplification. At the latest follow-up (85 months after surgery), the patient was alive, with no evidence of disease. To the best of our knowledge, this is the first example of a molecularly confirmed primary bladder liposarcoma and the first case of DDL at this site.

## 1 Introduction

Liposarcoma is one of the most common soft tissue sarcomas (STSs) among adults, accounting for approximately 15%–20% of all STSs (1). According to the latest World Health Organization (WHO) classification of soft tissue and bone tumors, liposarcoma is divided into four principal subtypes: atypical lipomatous tumor/well-differentiated liposarcoma/dedifferentiated liposarcoma (ALT/WDL/DDL), myxoid liposarcoma (ML), pleomorphic liposarcoma (PL), and myxoid pleomorphic liposarcoma (MPL). ALT/WDL/DDL has the highest incidence (>50%) and harbors specific genetic features—12q13-15 amplification, including *MDM2*, *CDK4*, *FRS2*, and *CPM*. In particular, *MDM2* is amplified in almost all cases of ALT/WDL/DDL, and detection of *MDM2* amplification by fluorescence *in situ* hybridization (FISH) has been recognized as the gold standard for the diagnosis of this tumor (2). The term “ALT” has been introduced to emphasize superficial soft tissue masses (2). WDL often occurs in central anatomic sites, such as the retroperitoneum and mediastinum, where tumors are more likely to be removed incompletely and are associated with frequent recurrence (2). DDL accounts for 18%–20% of liposarcomas and arises mostly in the retroperitoneum (3). Accounting for 20%–30% of liposarcomas, ML typically occurs in the extremities and is characterized by *FUS/EWSR1::DDIT3* fusion. PL represents less than 5% of liposarcomas and often arises in the extremities and trunk with complex chromosomal alterations. MPL, an emerging new subtype, is extremely rare and shows a predilection for the mediastinum in young patients.

Bladder sarcomas account for <5% of all bladder tumors (4). Primary liposarcoma arising from the urinary bladder is extremely rare, with only six cases reported in the English literature (5–10), all of which have been described as ML. Most importantly, all reported cases lacked genetic confirmation. Here, we report the first case of primary DDL of the urinary bladder, in which the dedifferentiated area exhibited a low-grade fibromatosis-like appearance. Notably, genetic alterations were identified using various methods in this case.

## 2 Methods

### 2.1 Immunohistochemistry

The specimens were formalin-fixed paraffin-embedded (FFPE) and cut into 4-μm sections for examination. Standard immunohistochemical staining was performed using the EnVision Plus detection system (DAKO, Carpinteria, CA, USA) with positive and negative controls. Information on the antibodies is presented in **Supplementary Table 1**.

### 2.2 Fluorescence *in situ* hybridization

The Vysis LSI *MDM2* Spectrum Orange Probe (Abbott Molecular, Des Plaines, IL, USA), GSP *CDK4* Gene Amplification Probe and *FRS2* Probe (Anbiping, Guangzhou, China) were used for *MDM2*, *CDK4*, and *FRS2* amplification. *ALK* rearrangement was performed using the Vysis LSI *ALK* Dual Color Break Apart Rearrangement Probe (Abbott Molecular, Des Plaines, IL, USA). FISH tests were performed according to an established protocol (11).

The sections were scored by two investigators and ≥100 cells were counted. Amplification was defined as an average *MDM2*/CEP12 ratio, *FRS2*/CEP12 ratio, and *CDK4*/CSP12 ratio ≥2.0, while a ratio <2.0 was considered non-amplified (11, 12). Positivity for *ALK* rearrangement was considered when ≥15% of nuclei displayed split signals or when single red signals were observed.

### 2.3 Detection of *CTNNB1* mutation

The primers for *CTNNB1* gene mutation polymerase chain reaction (PCR) detection were *CTNNB1*-F: 5’TCCAATCTACTAATGCTAATACTGTTTCGTA3’ and *CTNNB1*-R: 5’ TCCAATCTACTAATGCTAATACTGTTTCGTA3’. Sanger sequencing was performed by BGI Genomics Co., Ltd. (Shenzhen, China).

### 2.4 Whole-exome sequencing

WES was performed by Novogene Technology Co., Ltd. (Beijing, China) on FFPE tissues to explore possible molecular abnormalities.

## 3 Case presentation

**Figure 1:**
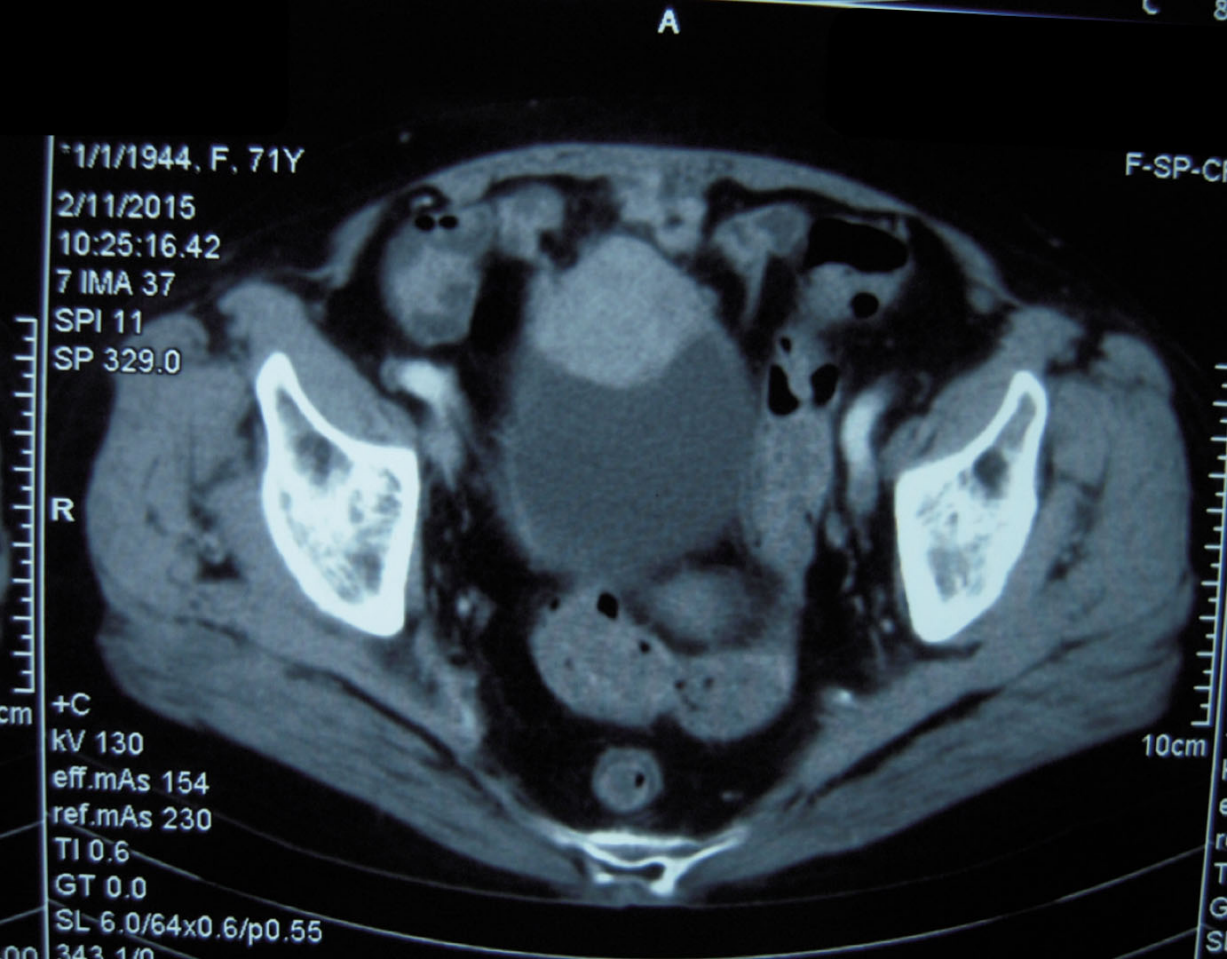
Abdominal computed tomography showed an irregular mass on the anterior wall of the bladder, which involved all layers of the bladder.

A 69-year-old female was admitted to our hospital with a one-month history of recurrent gross hematuria, accompanied by slight urgency and pain during urination. The patient had no history of trauma or infection. Physical examination revealed an approximately 5 cm × 4 cm palpable fixed smooth mass in the hypogastrium with no tenderness or rebound tenderness. Urine cytology revealed an increased number of leukocytes with no heterotypic cells or red blood cells. Computed tomography showed an ill-defined solid mass in the anterior wall of the bladder (**Figure 1**). During cystoscopy, the mucous membrane of the bladder wall appeared smooth with no evidence of novel organisms on the surface. Additionally, a bulge was identified at the bladder dome with a normal overlying urothelial mucosa. The biopsy indicated “chronic inflammation of the mucosa and lamina propria fibrous hyperplasia.” Subsequently, the patient underwent transurethral partial resection of the bladder tumor (TUR-BT). Despite no visible abnormalities in the bladder mucosa, a mass deep in the muscle layer was identified in the bladder dome during TUR-BT. Regrettably, the biopsy after TUR-BT still exhibited no signs of the tumor. After evaluation and communication with the patient, who expressed a strong desire to preserve the bladder, median laparotomy and bladder exploration were performed. During this surgery, a mass was identified adhering to the apex of the bladder, pubic symphysis, and peritoneum and was completely resected together with part of the bladder and peritoneum.

Macroscopic examination revealed a partially excised bladder measuring 7 cm × 7 cm × 5 cm. The overlying urothelium is rough. Thickening of the bladder wall was almost completely replaced by gray-white woven lesions, which showed diffuse infiltrative growth into perivesical soft tissues. On the cut surface, a gray-white and tough mass resembled the scar tissue.

**Figure 2:**
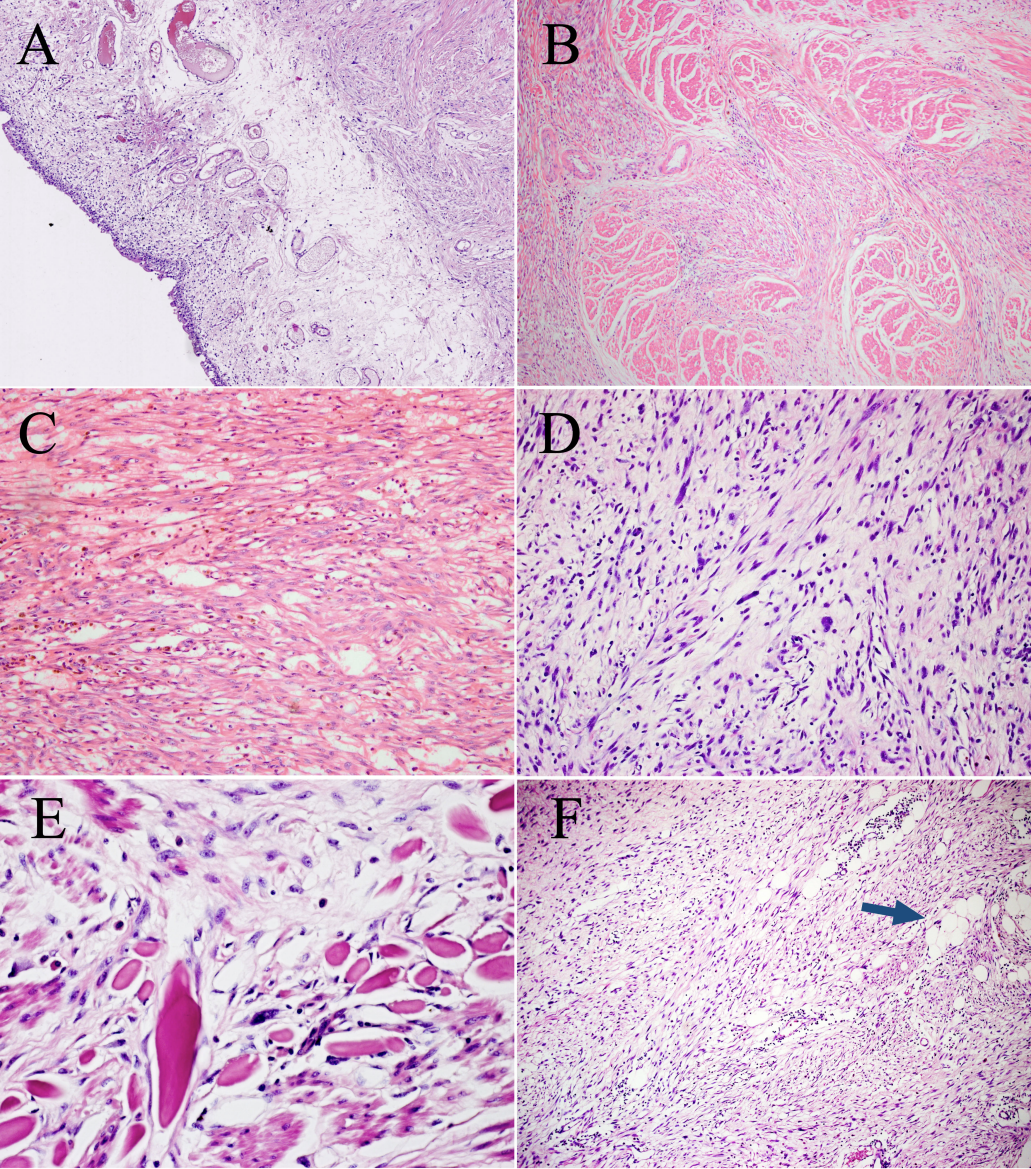
Microscopic features of the surgical resection sample. **(A)** The urothelium and lamina propria of the bladder show no abnormalities (H&E, ×100). **(B)** Bladder muscles were infiltrated to varying degrees by spindle cells. (H&E, ×100). **(C)** Pump spindle cells were mixed with a predominantly plasmacytic infiltrate (H&E, ×200). **(D)** The elongated spindle cells are arranged in a fascicular pattern (H&E, ×200). **(E)** Hyperchromatic tumor cells are observed within the background of spindle cells (H&E, ×400). **(F)** A few lipoma-like WDL components are identified (blue arrow, H&E, ×100).

Microscopically, the urothelium and lamina propria of the bladder showed no abnormalities (**Figure 2A**). The muscle layer of the bladder and peripheral adipose tissue contained spindle cells, which were arranged in a fascicular growth pattern with mild to moderate atypia and hyperchromatic nuclei, mimicking low-grade fibroblastic tumors (**Figure 2B**). Pump spindle and scattered inflammatory cells were observed in the superficial layer of the muscularis propria (**Figure 2C**). Additionally, in the deep layers of the muscularis propria and serosa, slender spindle cells arranged in a fascicular growth pattern were separated by abundant eosinophilic collagen (**Figure 2D**). Bizarre and hyperchromatic stromal cells were observed in these areas (**Figure 2E**). Notably, small foci of typical lipoma-like WDL components were identified within the background of the spindle cell tumor using extensive sampling (**Figure 2F**). The surgical cautery margin was negative and the peritoneal tissue was normal. This lesion was classified morphologically as grade 2 using the Fédération Nationale des Centers de Lutte Contre le Cancer (FNCLCC) grading system, and the postoperative staging was T1N0M0 according to the TNM staging of soft tissues tumors.

**Figure 3:**
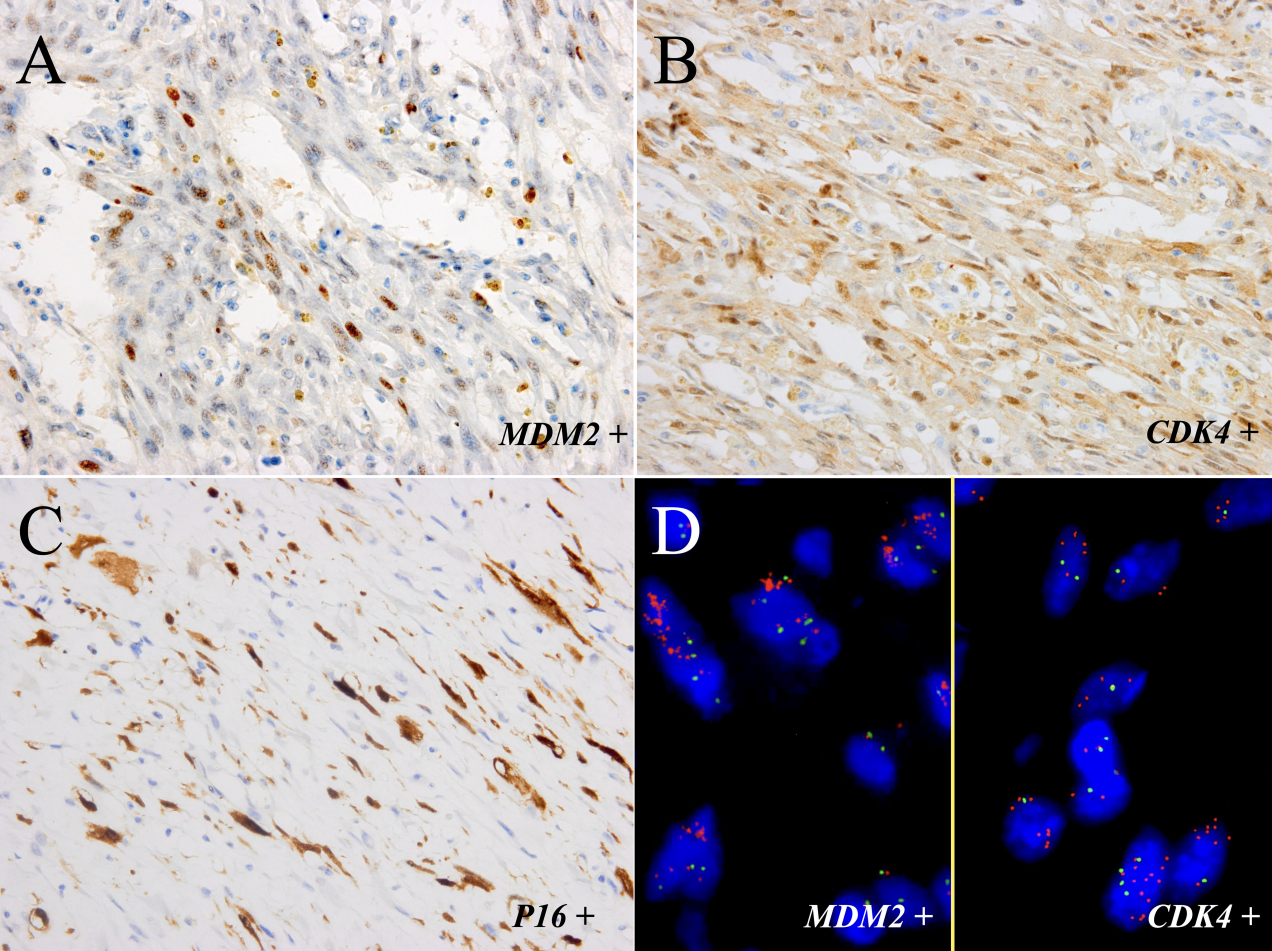
The tumor components of the sample were positive for MDM2 **(A)** and CDK4 **(B)** and focal positivity for P16 **(C)** (immunostaining, ×400). **(D)** FISH showed amplification of *MDM2* and *CDK4* in most neoplastic cells (left part: *MDM2*; right part: *CDK4;* red signals represent *MDM2* and *CDK4*, respectively. Green signals represent chromosome 12 centromeres (magnification: ×1000).

Immunohistochemically, the neoplastic cells were positive for MDM2 (**Figure 3A**), CDK4 (**Figure 3B**), P16 (**Figure 3C**), smooth muscle actin (SMA), and focal positivity for muscle-specific antigen (MSA). The MIB-1 index value was 5%. Tests for Desmin, ALK-1, p63, CK7, PCK, S-100, CD34, EMA, and β-catenin were negative. FISH revealed *MDM2* and *CDK4* amplification in the spindle cells and adipocytes of the tumor (**Figure 3D**), whereas negative results was observed in the adipose components surrounding the bladder wall. There was no evidence of *FRS2* amplification, *ALK* rearrangement, or *CTNNB1* mutation. Further WES showed *TSPAN31* and *JUN* amplifications.

These findings were consistent with the ultimate diagnosis of low-grade fibromatosis-like DDL arising from the urinary bladder. The patient did not receive any adjuvant therapy other than close follow-up for surveillance. The surveillance plan involved regular physical examination with imaging (chest and abdomen CT scan) and urine tests 1 month after the operation, every 3–6 months for 3 years, and then annually. During an 85-month follow-up period, the patient was alive with no evidence of disease recurrence.

## 4 Discussion

This study describes a case of primary liposarcoma arising from the urinary bladder. Bladder mesenchymal tumors are rare, representing 1%–5% of all primary urinary bladder tumors, and leiomyomas and leiomyosarcomas are the most common benign and malignant mesenchymal tumors of the bladder, accounting for approximately 0.43% and 1% of all bladder tumors, respectively (13, 14). Although liposarcoma is one of the most common soft tissue sarcomas, it rarely occurs in the bladder. Only six cases of bladder liposarcoma have been reported in the English literature (5–10). The clinicopathological features of all published cases are summarized in **Table 1**.

**Table 1:** Clinicopathological features of primary liposarcoma of the bladder in published English-language literatures.

Case No.	References (Published time)	Age/Sex	Subtype	Size (cm)	Symptoms/Preoperative Duration	IHC	Treatment	Outcome/Follow-up duration	Genetic result
1	Rosi et al. (1983) (5)	36/M	Myxoid	2.5	Hematuria/NA	NA	Partial cystectomy	ANED/30 months	ND
2	Widdison et al. (1989) (6)	57/F	Myxoid	3.5	Hematuria/1 year	NA	Partial cystectomy	ANED/3 months	ND
3	Kunze et al. (1994) (7)	76/M	Round cell	15.0	NA	NA	NA	NA	ND
4	Biernat et al. (1996) (8)	36/F	Myxoid	NA	NA	NA	NA	NA	ND
5	Delport et al. (2021) (9)	57/F	Myxoid/round cell	6.9	Macrohematuria/30 years	NA	Incomplete TURBT	ANED/24 month	ND
6	Chudal et al. (2021) (10)	26/F	Myxoid	21.7	Abdominal mass/1 month	NA	Surgery and chemotherapy	NA	ND
7	Current case	68/F	Dedifferentiated	3.0	Hematuria/1 month	MDM2 (+), CDK4 (+), P16 (+)	Partial cystectomy	ANED/85 months	*MDM2, CDK4, TSPAN31*, and *JUN* amplified

F, Female; M, Male; NA, not available; ND, not done;+ positive; -, negative; ANED, alive, no evidence of disease; DOD, dead of disease; IHC, Immunohistochemistry; TURBT, transurethral resection of bladder tumor.

All previously reported bladder liposarcomas were ML and round cell liposarcomas (high-grade ML). However, all published bladder ML studies lack genetic confirmation of the *FUS/EWSR1::DDIT3* fusion gene. In addition, ML occurs predominantly in the deep soft tissues of the extremities (especially the thigh) and rarely occurs in other sites, including the retroperitoneum. Previous studies proposed that there was no existence of true primary retroperitoneal ML, and these “retroperitoneal ML” were proven as metastasis or DDL with myxoid-like changes (15, 16). Only 2.3% (5/214) of liposarcomas in the retroperitoneum were identified as primary ML by the detection of *DDIT3* arrangement in a large series study (17). Owing to the rarity of primary retroperitoneal ML, molecular genetic validation and examinations to exclude metastasis are required to confirm the primary retroperitoneal origin. Thus, it is possible that some of these reported bladder liposarcomas are not real primary ML. Some cases could be explained by the presence of myxoid-like stroma in sarcomas such as DDL, MPL, and undifferentiated sarcomas. Therefore, further studies with a larger series are needed to reveal whether there are unique features of liposarcomas occurring in the urinary bladder.

Notably, the current case exhibited peculiar low-grade fibroblastic tumor-like morphological features. DDL has a wide morphological spectrum, and most dedifferentiated areas exhibit an intermediate- to high-grade histological morphology, resembling undifferentiated pleomorphic sarcoma or myxofibrosarcoma. DDLs with low-grade differentiation have increasingly been recognized in recent years, and these dedifferentiated areas are histologically similar to low-grade myofibroblastic sarcoma, fibromatosis, or inflammatory myofibroblastic tumors (IMT) (18). Besides, this present lesion exhibited “pure” low-grade morphology. There are a limited number of cases of DDL entirely composed of low-grade dedifferentiated components, as low-grade and high-grade dedifferentiated areas commonly coexist.

The genetic results of this case, especially the amplification of genes in the 12q13-15 region, strongly supported the final diagnosis of DDL. However, all previously reported cases of bladder liposarcoma have lacked genetic confirmation. The current case is the first liposarcoma in the urinary bladder that was validated by molecular methods. Additionally, the results were negative for *FRS2* amplification. *FRS2* is located close to *MDM2* within the 12q13-15 region and is amplified in >90% of ALT/WDL/DDL and low-grade osteosarcoma cases (19, 20). Jing et al. (19) summarized 146 consecutive cases of ALT/WDL/DDL and identified 10 cases of *MDM2*(+)/*FRS2*(−) ALT/WDL/DDL (6.8%; including seven ALT/WDLs and three DDLs). All three previously reported *MDM2*(+)/*FRS2*(−) DDLs were in peripheral sites, and their dedifferentiation components included two homologous pleomorphic liposarcoma-like and one intermediate-grade fibrosarcoma-like. The present case represents the first case of *MDM2*(+)/*FRS2*(−) DDL located in a central site with a low-grade dedifferentiated area. Furthermore, amplification of the *JUN* gene was identified in this lesion. Outside of 12q13-15, amplification of 1p32 (including *JUN*) and 6q23 (including *ASK1*) was observed in both ALT/WDL and DDL but was more correlated with undifferentiated histology (21–23). The detection of *JUN* amplification in this tumor could further assist in the diagnosis of the DDL subtype.

The overlapping clinical and histological features between this case and several spindle cell lesions resulted in diagnostic dilemmas (24). Misdiagnosis can easily occur, particularly when well-differentiated components are obscured. Differential diagnoses in this case included secondary DDL, cellular ALT/WDL, bladder sarcomatoid carcinoma (SC), and other more common bladder mesenchymal tumors, such as IMT, desmoid-type fibromatosis (DF), and rhabdomyosarcoma (RMS).

Owing to the rarity of bladder liposarcoma, the possibility of secondary DDL should first be excluded. However, the patient did not have a history of liposarcoma, and imaging examinations did not reveal any evidence of a tumor other than the bladder. More importantly, the distinction of *MDM2* amplification between mass and adipose tissue outside the bladder wall suggested that the tumor originated from the bladder.

Cellular ALT/WDL and DDL share overlapping histological features and common genetic alterations. However, cellular ALT/WDL is partly composed of adipocytic proliferation and contains atypical adipocytes or lipoblasts, whereas dedifferentiated areas of DDL are usually non-lipogenic. The overwhelming rate of fibroblastic tumor-like histology and hyperchromatic stromal cells with moderate atypia in this case was favorable for the diagnosis of DDL. The survival of DDL was shorter than that of cellular ALT/WDL in a study by Evans et al. (25). Although there were no signs of recurrence or metastasis in the current case, regular close follow-up is necessary.

SC would be indistinguishable from liposarcoma when SC with a heterologous liposarcoma component. However, only three cases of bladder SC with a liposarcomatous component have been reported (26–28). It should be noted that no carcinomatous component was identified, whereas a lipoma-like WDL component was found by wide sampling in our case. Combined with the negativity for epithelial markers and the amplification of several genes in 12q13-15 in this case, the diagnosis of DDL was more appropriate.

IMT, DF, and RMS appear to be the most common tumor types in the urinary bladder. Moreover, the dedifferentiated area in this case exhibited peculiar low-grade fibroblastic tumor-like morphological features mimicking IMT and DF. However, the identification of a typical lipoma-like WDL component, along with the absence of *ALK* rearrangement and the negativity of β-catenin and *CTNNB1* mutations, did not support the diagnosis of IMT and DF, respectively (29, 30). RMS mainly occurs in children and adolescents, is characterized by small primary round cells, and usually exhibits a high rate of mitotic activity, which differs from the demonstration in this case. Most importantly, detection of *MDM2* gene amplification by FISH in this lesion further confirmed the correct diagnosis of DDL.

Imaging, cystoscopy, and histopathological diagnosis with cystoscopy biopsy and TUR-BT were performed in this patient to comprehensively evaluate and opt for the optimum treatment. However, cystoscopy and TUR-BT biopsies were both too shallow to reach the muscle layer of the bladder where the tumor was located. The patient ultimately underwent partial bladder resection rather than a cystectomy. This consideration was made in view of the patient’s desire to retain the bladder and the identification that the tumor was confined to the bladder during surgery, and the surrounding tissue was removed as much as possible to achieve a negative margin. Although the patient did not undergo postoperative chemoradiotherapy due to personal choice, close and regular follow-up ensured that any tumor progression could be managed promptly. Notably, both *MDM2* and *CDK4* were amplified in this tumor, which could provide an opportunity for the patient to benefit from targeted therapy. *CDK4* inhibitors, such as palbociclib, have recently been approved for clinical treatment of *CDK4*+ liposarcoma (31). *MDM2* is the most important driver gene of ALT/WDL/DDL, and although it is still being tested in clinical trials, it holds promise as an effective treatment option, especially for patients with disease recurrence or tumor metastasis (32).

Notably, this case had a favorable prognosis, and no recurrence or metastasis was observed 85 months after surgery. Extensive surgical resection with negative margins may be the main reason (33). Additionally, although malignant, DDL generally has a relatively better prognosis than most sarcomas, and the lesion is morphologically low-grade. Studies have indicated that low-grade DDL has longer survival times than high-grade DDL (34). Several studies have consistently demonstrated that increased *CDK4* amplification is strongly correlated with poor outcomes, and *CDK4* amplification levels are significantly higher in high-grade DDL than in low-grade DDL (23, 35). Despite the need for more studies based on large series for validation, the *MDM2*(+)/*FRS2*(−) genotype in this case has been reported to be associated with indolent clinical behavior (19, 20). Nevertheless, the current case harbored *JUN* amplification, which has been reported to be related to shorter recurrence-free survival time and poor prognosis in ALT/WDL/DDL (23). However, in that previous study, the comparison between amplification and non-amplification groups did not rule out the effect of subtype while *JUN* amplification has been known to be associated with DDL, which represents the subtype with a poorer outcome. The relationship between *JUN* amplification and liposarcoma prognosis requires further study. Notably, anatomical location is the most important prognostic factor for liposarcoma, and retroperitoneal liposarcomas generally exhibit the worst clinical behavior (2). This implies that bladder DDL have a poor prognosis. Although this case has shown a good prognosis during the follow-up period, it has been reported that almost all retroperitoneal DDLs recur locally if the follow-up duration is longer than 10 years (2). Therefore, the follow-up time in this specific case may have been insufficient. More cases and further extensive research are required to reveal the prognosis of bladder liposarcoma.

In conclusion, we report an exceedingly rare case of primary low-grade fibroblastic tumor-like DDL occurring in the urinary bladder, which should be the first genetically confirmed example using various methods. Additional cases and further studies are needed to explore the clinicopathological features of bladder liposarcomas. For cases arising from rare sites, careful histological inspection and rational application of molecular detection can be valuable for making a correct diagnosis.

## Data availability statement

The original contributions presented in the study are included in the article/**Supplementary Materials**. Further inquiries can be directed to the corresponding author.

## Ethics statement

The studies involving humans were approved by the Ethics Committee for Research in Human Beings of West China Hospital of Sichuan University. The studies were conducted in accordance with the local legislation and institutional requirements. The participants provided their written informed consent to participate in this study. Written informed consent was obtained from the individual(s) for the publication of any potentially identifiable images or data included in this article. Written informed consent was obtained from the participant/patient(s) for the publication of this case report. Written informed consent was obtained from the participant/patient(s) for the publication of this case report.

## Author contributions

JC and RP analyzed the data and prepared the manuscript. YZ collected the clinicopathological data of the patient. YL and MC helped with molecular experiments. XH helped histopathology review. HZ was responsible for the diagnosis, study design and the manuscript revision. All authors contributed to the article and approved the submitted version.
