# Supplementary material for: Case Report: Primary low-grade dedifferentiated liposarcoma of the urinary bladder with molecular confirmation
Source: Front Oncol. 2023 Oct 10;13:1221027. doi: 10.3389/fonc.2023.1221027 (PMC10597670; doi:10.3389/fonc.2023.1221027)
Supplement: Supplementary file 1 [file Table_1.docx]

Supplementary Material

Case Report: Primary Low-grade Dedifferentiated Liposarcoma of the Urinary Bladder with Molecular Confirmation

Jian Cui^1†^, Ran Peng^1†^, Yahan Zhang^1^, Yang Lu^1^, Xin He^1^, Min Chen^1^, and Hongying Zhang^1^^*^

^1^ Department of pathology, West China Hospital, Sichuan University, Chengdu, China

^†^ These authors have contributed equally to this work

*** Correspondence:** Hongying Zhang

hy_zhang@scu.edu.cn;

hy_zh@263.net

# Supplementary table 1. The information of antibodies used in immunohistochemistry

| **Antibody** | **Clone** | **Dilution** | **Manufacturers** |
| --- | --- | --- | --- |
| smooth muscle actin | 1A4 | 1:100 | Dako, Carpinteria, CA, USA |
| MDM2 | SMP14 | ready-to-use | Abcam, Cambridge, UK |
| CDK4 | EP180 | 1:100 | Santa Cruz, CA, USA |
| p16 | 16P04/JC2 | ready-to-use | NeoMarkers, Fremont, CA, USA |
| muscle specific antigen | HHF35 | 1:50 | Abcam, Cambridge, UK |
| desmin | D33 | 1:100 | Dako, Carpinteria, CA, USA |
| anaplastic lymphoma kinase (ALK-1) | 5A4 | 1:150 | Abcam, Cambridge, UK |
| p63 | 4A4 | 1:100 | Abcam, Cambridge, UK |
| cytokeratin | AE1/AE3 | 1:200 | SantaCruz, CA, USA |
| S-100 protein | 4C49 | 1:100 | Abcam, Cambridge, UK |
| CD34 | QBEnd 10 | 1:100 | Abcam, Cambridge, UK |
| epithelial membrane antigen | E29 | 1:100 | ScyTek, Logan, UT, USA |
| β–catenin | EP35 | ready-to-use | Alton Pkwy Irvine, CA, USA |
| ki-67 | MIB-1 | 1:100 | Abcam, Cambridge, UK |
